# Could high serum folate be associated with adverse effects?

**DOI:** 10.22038/IJBMS.2020.51870.11762

**Published:** 2020-12

**Authors:** Mahla Ghorbani, Javad Baharara, Mohammad Amin Kerachian

**Affiliations:** 1 Department of Biology, Faculty of Sciences, Mashhad Branch, Islamic Azad University, Mashhad, Iran; 2 Research Center for Animal Development Applied Biology & Biology Department, Mashhad Branch, Islamic Azad University, Mashhad, Iran; 3 Medical Genetics Research Center, Mashhad University of Medical Sciences, Mashhad, Iran.; 4 Department of Medical Genetics, Faculty of Medicine, Mashhad University of Medical Sciences, Mashhad, Iran; 5 Cancer Genetics Research Unit, Reza Radiotherapy and Oncology Center, Mashhad, Iran

**Keywords:** Agouti, Epigenetic, Folic acid, Methylation, MTHFR, Pregnant

## Abstract

Folate is an important water-soluble vitamin that is presented naturally in foods in particular vegetables, fruits, and whole grains. To which extent is this vitamin needed in our daily regimen is not fully known. Several studies have indicated that many complications, such as megaloblastic anemia, cardiovascular disease, neural tube defects, and numerous cancers, occur in humans when the body becomes deficient in folic acid. On the other hand, a few studies have shown thier concerns regarding the supplementation of folic acid, resulting in the development of existing tumors and alteration of normal patterns of DNA methylation. Although there is no clear evidence of aberrant DNA methylation and gene expression changes in response to “high” levels of folate or folic acid intake, there are still some concerns. Therefore, its adverse effects especially on fetus and later stages of life should be carefully investigated.

## Introduction

Folate is an essential water-soluble vitamin found naturally in selected foods such as leafy green vegetables, brown rice, and granary bread. In its synthetic form (folic acid), it is used in supplements and in food fortified foods like breakfast cereals (1). To which extent is this vitamin needed in our daily regimen is still questionable. Animal studies have indicated that folic acid could improve both short- and long-term memories, in a dose-dependent manner (2) and has a therapeutic and preventive effect on cognitive impairments in Alzheimer’s disease (3). 

On the other hand, when the human body becomes deficient in folic acid, many problems such as megaloblastic anemia, cardiovascular disease, neural tube defects (NTD) and multiple cancers occurs (4). The mechanisms by which low folate status contribute to these disorders has remained unclear, although several molecular pathways for the function of folate have been identified so far. Folate is recognized to have critical roles in the synthesis of DNA, and the modification of DNA/RNA.

In the intestine, folate is reduced to dihydrofolate by dihydrofolate reductase and subsequently to tetrahydrofolate (THF) and converted to 5,10-methylene THF by the vitamin B6 dependent enzyme serine hydroxymethyltransferase. Afterwards it is reduced to 5-methyl THF by methylenetetrahydrofolate reductase (MTHFR). This is important to transfer methyl groups for the remethylation of homocysteine to methionine through the vitamin B_12_ dependent methionine synthase reaction. Methionine is the substrate for a methyl group donor causing numerous methylation reactions in the body (5). 

In the aspect of pathogenesis, the homozygosity for the T allele of the C677T polymorphism of MTHFR could be a risk factor for several diseases. Both the homozygous (TT) and heterozygous (CT) genotypes are associated with lower tissue concentrations of folate, higher homocysteine concentrations, and lower enzyme activity than the wild type (CC) genotype. It has been shown that low folate and higher homocysteine levels in early pregnancy are risk factors for NTB. Both the lower folate and increased homocysteine concentrations associated with CT and TT genotypes can be corrected by folic acid administration, even in relatively small doses (6). Thus, it is recommended that all women who could get pregnant should take a daily supplement of 400 micrograms of folic acid prior to pregnancy and also during the first 12 weeks of gestation, at the time the fetus’ spine is developing. Some women are advised to take a higher dose of 5 milligrams of folic acid each day until they are 12 weeks pregnant if they have a higher chance of having a pregnancy affected by NTD; that is a family history of NTD, a previous affected child, diabetes and a drug history intake of anti-epilepsy medicine. Although the folic acid could prevent from NTD, the adverse effect of overdoses of folic acid in fetus is not fully studied. Therefore, there is a possibility to have a number of impacts later in life. 

Several studies demonstrated thier concerns about folic acid supplementation resulting in progression of existing tumors and altering normal DNA methylation patterns (7). Although there is no direct evidence of aberrant DNA methylation and changes in gene expression in response to “high” levels of folate/folic acid intake, concerns still exist for the adverse impact of folate (1). 

In experimental animals several effects of folic acid has been reported. For example, in the Agouti mouse model the importance of maternal nutrition in shaping the epigenome of offspring has been investigated. The abundance of methyl donors in the maternal diet, such as folic acid and S-adenosyl methionine, determines the methylation of the Agouti gene, which in turn renders the mouse’s coat yellow and dark when it is hypomethylated and methylated, respectively (8). The yellow-colored mice have a predisposition to obesity and the development of cancer. Interestingly, a maternal diet rich in methyl donors has been shown to overcome the negative effects of in utero bisphenol A (BPA) exposure. BPA was demonstrated to lead to global hypomethylation of the offspring epigenome, but supplementation of the maternal diet with folic acid, betaine, vitamin B_12_, choline, or genistein was found to neutrilze these effects of BPA (9). Although these experiments or similar studies have been focused on the positive effects of methyl donor agents especially folic acid, the negative influences should not be dismissed. At this time, there are insufficient data to determine whether there is an effect of higher doses of folic acid at any particular locus, genomic region, specific tissue type, or developmental state and whether the change would result in increased risk or benefit. Only in a recent study by Hoyo *et al*., they examined the DNA methylation level in the cord blood of newly delivered infants at DNA sequences regulating IGF2 expression and found significantly decreased DNA methylation level (toward the expected 50% level) with increasing maternal folic acid intake in pregnancy (10). This study was very limited by its small sample size. 

## Conclusion

Although folic acid, a leading methyl donor, is crucial for many biological reactions in our body its adverse effects especially on fetus and later stages of life especially on high doses should be thoroughly investigated.
